# A comprehensive review of transcranial magnetic stimulation in secondary dementia

**DOI:** 10.3389/fnagi.2022.995000

**Published:** 2022-09-26

**Authors:** Giuseppe Lanza, Francesco Fisicaro, Raffaele Dubbioso, Federico Ranieri, Andrei V. Chistyakov, Mariagiovanna Cantone, Manuela Pennisi, Alfio Antonio Grasso, Rita Bella, Vincenzo Di Lazzaro

**Affiliations:** ^1^Department of Surgery and Medical-Surgical Specialties, University of Catania, Catania, Italy; ^2^Clinical Neurophysiology Research Unit, Oasi Research Institute-IRCCS, Troina, Italy; ^3^Department of Biomedical and Biotechnological Sciences, University of Catania, Catania, Italy; ^4^Department of Neurosciences, Reproductive Sciences and Odontostomatology, University of Naples “Federico II”, Naples, Italy; ^5^Unit of Neurology, Department of Neuroscience, Biomedicine and Movement Sciences, University of Verona, Verona, Italy; ^6^Laboratory of Clinical Neurosciences, Rambam Medical Center, Haifa, Israel; ^7^Neurology Unit, Policlinico University Hospital “G. Rodolico – San Marco”, Catania, Italy; ^8^Neurology Unit, Sant’Elia Hospital, ASP Caltanissetta, Caltanissetta, Italy; ^9^Department of Medical and Surgical Sciences and Advanced Technologies, University of Catania, Catania, Italy; ^10^Unit of Neurology, Neurophysiology and Neurobiology, Department of Medicine and Surgery, Università Campus Bio-Medico di Roma, Rome, Italy; ^11^Fondazione Policlinico Universitario Campus Bio-Medico, Rome, Italy

**Keywords:** transcranial magnetic stimulation, dementia, cortical excitability, cognitive impairment, electrophysiology, neuromodulation

## Abstract

Although primary degenerative diseases are the main cause of dementia, a non-negligible proportion of patients is affected by a secondary and potentially treatable cognitive disorder. Therefore, diagnostic tools able to early identify and monitor them and to predict the response to treatment are needed. Transcranial magnetic stimulation (TMS) is a non-invasive neurophysiological technique capable of evaluating *in vivo* and in “real time” the motor areas, the cortico-spinal tract, and the neurotransmission pathways in several neurological and neuropsychiatric disorders, including cognitive impairment and dementia. While consistent evidence has been accumulated for Alzheimer’s disease, other degenerative cognitive disorders, and vascular dementia, to date a comprehensive review of TMS studies available in other secondary dementias is lacking. These conditions include, among others, normal-pressure hydrocephalus, multiple sclerosis, celiac disease and other immunologically mediated diseases, as well as a number of inflammatory, infective, metabolic, toxic, nutritional, endocrine, sleep-related, and rare genetic disorders. Overall, we observed that, while in degenerative dementia neurophysiological alterations might mirror specific, and possibly primary, neuropathological changes (and hence be used as early biomarkers), this pathogenic link appears to be weaker for most secondary forms of dementia, in which neurotransmitter dysfunction is more likely related to a systemic or diffuse neural damage. In these cases, therefore, an effort toward the understanding of pathological mechanisms of cognitive impairment should be made, also by investigating the relationship between functional alterations of brain circuits and the specific mechanisms of neuronal damage triggered by the causative disease. Neurophysiologically, although no distinctive TMS pattern can be identified that might be used to predict the occurrence or progression of cognitive decline in a specific condition, some TMS-associated measures of cortical function and plasticity (such as the short-latency afferent inhibition, the short-interval intracortical inhibition, and the cortical silent period) might add useful information in most of secondary dementia, especially in combination with suggestive clinical features and other diagnostic tests. The possibility to detect dysfunctional cortical circuits, to monitor the disease course, to probe the response to treatment, and to design novel neuromodulatory interventions in secondary dementia still represents a gap in the literature that needs to be explored.

## Introduction

### Background

Primary dementia typically stems from a progressive and degenerative/irreversible neuronal loss, whereas secondary forms are characterized by a progressive, but potentially reversible, cause of cognitive decline. Common examples of primary dementias are Alzheimer’s disease (AD), dementia with Lewy bodies (DLB), fronto-temporal lobar degeneration (FTD), Huntington’s disease (HD), and Creutzfeldt-Jakob’s disease. Among conditions that may cause or mimic dementia, which usually affects younger subjects than primary dementia (Sobów et al., 2007), there are normal-pressure hydrocephalus (NPH), multiples sclerosis (MS), celiac disease (CD) and other immunologically mediated diseases, as well several inflammatory, infective, metabolic, toxic, nutritional, endocrine, hepatic, renal, sleep-related, and rare genetic disorders (Yousuf et al., 2010).

The most relevant aspect of all these conditions is that, if cognitive impairment is early detected and promptly treated, it can be reversed or its course significantly improved, even toward a complete recovery (Lowenthal et al., 2007; Pennisi et al., 2020; Caruso et al., 2022). In this context, a systematic neurophysiological assessment of secondary dementia can aid the diagnosis, follow the course, and predict the response to treatment. Among electrophysiological techniques, transcranial magnetic stimulation (TMS) has emerged as a valuable non-invasive method for the functional evaluation *in vivo* and in “real time” of the cortico-spinal tract and cortico-cortical connections, capable of revealing neurotransmission abnormalities in several disorders causing cognitive impairment and dementia (Rossini et al., 2007; Cantone et al., 2014; Di Lazzaro et al., 2021).

### Transcranial magnetic stimulation

From pioneering applications of TMS to assess the primary motor cortex (M1) functioning and the cortical-spinal tract (Barker et al., 1985; Cantone et al., 2019b; Lanza et al., 2019a, 2020b), researchers have boosted the potentialities of this technique, which is today employed to study cortical excitability, to map connectivity (Dubbioso et al., 2021), and to probe distinctive plastic phenomena (Rothwell, 2018). Overall, this has provided insights into the pathophysiology, treatment, and monitoring of different neurological and neuropsychiatric disorders (Di Pino et al., 2014a; Gomes-Osman et al., 2018; Cantone et al., 2019a).

A functional evaluation of the cortical excitability and cortico-spinal conductivity is performed by applying single-pulse TMS at proper stimulation intensity on the M1, thus eliciting motor evoked potentials (MEPs) that can be recorded from selected muscles of the contralateral side (Di Lazzaro et al., 2001; Hoogendam et al., 2010). The amplitude of MEP expresses a compound index of the output cells excitability within the M1, motor axons, nerve roots, peripheral motor nerves, and muscles (Rossini et al., 2015). The resting motor threshold (rMT), i.e., the minimal stimulation intensity required to elicit MEPs, is considered as a global measure of cortical excitability, since it is an aggregate index of the excitability of motoneuron membranes, neuronal inputs in cortical pyramidal neurons, spinal motoneurons, neuromuscular junctions, and muscle cells (Rossini and Rossi, 2007). Central motor conduction time (CMCT), estimated by subtracting the conduction time along the peripheral nerve (after stimulation of the motor roots) from the MEP cortical-muscular latency, measures the conduction along the cortico-spinal tract (Rossini et al., 2015).

The cortical silent period (cSP), which refers to the electromyographic inhibition of the motor responses occurring after a suprathreshold stimulation of the M1 during a tonic voluntary contraction of contralateral muscles, is an index of the inhibitory intracortical circuit (Chen et al., 1999). This parameter, which normally lasts a few hundred ms, is considered as a dynamic metric of a specific inhibitory intracortical circuitry (Cantello et al., 1992; Werhahn et al., 1999), largely mediated by the gamma-aminobutyric acid (GABA)-B activity (Siebner et al., 1998). The muscle activation and hemisphere stimulation of the same side evokes the ipsilateral SP (iSP), which it is thought to be generated by transcallosal fibers projecting into contralateral GABAergic interneurons (Kobayashi and Pascual-Leone, 2003; Chen et al., 2008; Lanza et al., 2013).

Paired-pulse TMS paradigms allow the assessment of the short-interval intracortical inhibition (SICI) and the intracortical facilitation (ICF) phenomena (Kujirai et al., 1993; Ziemann et al., 1996; Faro et al., 2010). The activity of GABA-A interneurons is the most likely mediator of SICI (Di Lazzaro et al., 2006a; Ferreri et al., 2011), whereas the neurophysiology of ICF is more complex. It probably relates to the activation of a cortical circuit projecting upon cortico-spinal cells different from that preferentially activated by single-pulse TMS. ICF seems to be greatly mediated by excitatory glutamatergic interneurons (Ziemann, 2004; Di Lazzaro et al., 2006c).

Additionally, the short-latency afferent inhibition (SAI) is known to largely reflect central cholinergic activity (Tokimura et al., 2000). Accordingly, in normal conditions, scopolamine (a muscarinic antagonistic) abolishes or reduces SAI (Di Lazzaro et al., 2000), whereas acetylcholine positively stimulates it (Di Lazzaro et al., 2005). Previous evidence suggests that SAI might reflect the integrity of intracortical circuitries underlying sensory-motor integration (Sailer et al., 2003; Martorana et al., 2009; Dubbioso et al., 2017b). SAI provides useful insights in several disorders affecting cognition and movement (Di Lazzaro et al., 2006b,^2007^; Dubbioso et al., 2019). TMS can also assess the mechanisms underlying synaptic plasticity by means of the paired-associative stimulation (PAS), through the application of magnetic stimuli after a short time interval of exercise or by means of low-frequency repetitive electric stimulation of median nerve coupled with TMS of the contralateral M1 (Carson and Kennedy, 2013). PAS leads to long-term potentiation (LTP)- and long-term depression (LTD)-like phenomena.

Finally, repetitive TMS (rTMS) applied on the same area of the cortex is able to induce temporary changes of cortical excitability, that is suppressed by low frequency stimulations (≤1 Hz) and enhanced by high frequencies (>5 Hz) of stimulation (Chen, 2020). Neurobiological phenomena underlying rTMS share features with the induction of LTP and LTD effects after tetanic stimulation of cortical slices (Wilson et al., 2018), including the correlation with the activity of the *N*-methyl-D-aspartate (NMDA) receptor (Cheeran et al., 2010), the sensitivity to earlier synaptic activation (Jung and Ziemann, 2009), and the strict link with stimulation frequency (Di Lazzaro et al., 2010). The short-term change in synaptic efficacy and the rapid down-regulation of GABA-related inhibitory circuits are key processes of the calcium- and sodium channel-dependent LTP plasticity (Ziemann et al., 1998a,b). Conversely, by inducing LTD-like changes, rTMS decreases synaptic efficacy (Gladding et al., 2009; Cirillo et al., 2017), both in normal and in pathological conditions (Fisicaro et al., 2020a). The effects of repeated sessions of rTMS persist in time and act by enhancing plasticity or by down-regulating it when it appears inappropriate or even maladaptive (Lefaucheur, 2019). For this reason, translational therapeutic and rehabilitative applications of rTMS can encompass a considerable number of neurological and neuropsychiatric diseases (Lefaucheur et al., 2020; Concerto et al., 2022). Additionally, distinctive rTMS protocols, such as the theta burst stimulation (Li et al., 2019) and quadripulse stimulation (Matsumoto and Ugawa, 2020), can aid toward a further understanding of the complex phenomena of neural plasticity, including the so-called metaplasticity (the “plasticity of synaptic plasticity”) (Di Pino et al., 2014b; Yger and Gilson, 2015; He et al., 2020; Cantone et al., 2021).

Transcranial magnetic stimulation and rTMS protocols are well tolerated and safe. Relatively frequent side effects that have been reported during or after rTMS are transient headache and a discomfort secondary to facial or scalp muscular twitching (Burt et al., 2002), whereas seizure induction represents a very rare adverse event (Anderson et al., 2006). As such, patients with epilepsy or those at risk for epilepsy need to be cautiously managed.

### Rationale and aim

Even if not always clinically evident, the involvement of motor areas in dementia has been demonstrated by neuropathological, and neuroimaging studies. The changes in motor areas might be caused by the disease process itself or, more often, might be caused indirectly as a consequence of remodeling mechanisms (Cantone et al., 2014). Indeed, increasing evidence supports the involvement of brain plasticity phenomena in different types of dementia, related to functional and structural changes within a very complex framework (Ferreri and Rossini, 2013).

To date, the implications of the cortical excitability changes observed in dementia are not completely understood. Moreover, while an increasing number of evidences is accumulating over time in AD, in other degenerative dementias (Cantone et al., 2014), and in vascular dementia (VaD) (Cantone et al., 2020), to date a comprehensive summary of the TMS findings in all other dementing processes is lacking. Here, we provide an up-to-date review of available studies, including data about pathophysiology, diagnosis, prognosis, and neuromodulatory interventions.

## Methods

A literature search was carried out to find all the relevant studies on TMS and rTMS in secondary dementia or cognitive impairment. A PubMed-based literature review was performed, from database inception to July 14, 2022, by using the search terms “secondary dementia” OR “secondary cognitive impairment” OR “reversible dementia” OR “reversible cognitive impairment” AND “normal pressure hydrocephalus” OR “multiple sclerosis” OR “celiac disease” OR “immune-mediated” OR “endocrine” OR “hepatic” OR “renal” OR “metabolic” OR “nutritional” OR “alcohol” OR “infectious” OR “inflammatory” OR “sleep” OR “genetic” AND “transcranial magnetic stimulation” OR “motor evoked potential” OR “motor cortical excitability.”

Two authors (F.F. and M.C.) independently screened titles and abstracts of the retrieved publications, and disagreements were solved by a third author (M.P.). Articles listed in the references were also reviewed in search of more data. Duplicated entries, retracted publications, studies on other diseases, works on animals or *in vitro*, non-English written papers, publications that are not research studies (e.g., commentaries, letters, editorials, reviews, etc.), and any other article that did not fit with the primary scope of this review (e.g., intellectual disability and other childhood-onset developmental-related cognitive deficits) were excluded. In particular, patients with vascular cognitive impairment (from mild form to overt VaD) were not included since, although some of them may present a more favorable course and even be partially reversible, to date there is no definitive consensus in the literature concerning the reversibility of this etiology. Moreover, both TMS and rTMS studies in vascular cognitive impairment and its subtypes (including VaD) have been recently reviewed (Cantone et al., 2020; Di Lazzaro et al., 2021). The same holds true for cognitive disorders due to psychiatric diseases (particularly depression) and neurosurgical conditions (e.g., traumatic brain injury, subdural hematoma, brain tumors, etc.).

## Results

A total of 475 items were originally retrieved. Of these, 79 peer-reviewed publications were selected according to the inclusion and exclusion criteria and the main findings analyzed clustering within two groups: one on TMS (Table 1) and the other on rTMS studies (Table 2).

**TABLE 1:** Relevant transcranial magnetic stimulation (TMS) findings reported in most common secondary cognitive disorders.

References	N.	Age, years (range)	Disease	rMT	CMCT	SAI	SICI	ICF	cSP	iSP	LTP
Zaaroor et al., 1997	24 patients 16 controls	72.0 (49.0–82.0) 63.5 (30.0–67.0)	NPH		↑ in non- responders						
Chistyakov et al., 2012	23 patients 8 controls	75.0 ± 7.8 (60.0–92.0) 74.8 ± 4.1 (71.0–84.0)	NPH	↓	=		↓	=			
Nardone et al., 2019a	20 patients 20 controls	73.6 74.1	NPH	↓		↓	↓				
Agrawal et al., 2021	11 patients 13 controls	69.0 ± 6.7 54.4 ± 4.4	NPH	↓ after drainage	↑		↓	=	=	↑	
Cucurachi et al., 2008	20 patients 20 controls	43.9 ± 10.7 NR	MS	=		↓					
Mori et al., 2011	42 patients 12 controls	23.0–54.0 25.0–52.0	MS	=			=, also LICI	=			↓
Mori et al., 2013	80 patients	17.0–39.0	MS	=			=, also LICI	=			↓pre = after therapy
Canham et al., 2015	28 patients	51.9 (34.9–70.0)	MS		↑						
Hofstadt-van Oy et al., 2015	17 patients 48 controls	39.72 ± 10.33 30.54 ± 8.50	MS								
Nantes et al., 2016	30 preserved functions 13 impaired functions 29 controls	42.9 ± 11.0 48.6 ± 12.6 45.0 ± 13.0	MS	=			=		↑		
Chalah et al., 2018	50 patients	51.82 ± 12.72	MS							↓	
Chalah et al., 2019	21 fatigued 17 non-fatigued	53.0 (34–62) fatigued 51.0 (44–67) non-fatigued	MS	=			↓at 2 ms	=	=	=	
Chaves et al., 2019	82 patients	47.51 ± 10.2	MS	=							
Chaves et al., 2021b	110 patients	48.4 ± 10.5	MS	↑	↑				↑	↑	
Chaves et al., 2021a	110 patients	47.59 ± 10.64 (males = 30) 48.64 ± 10.51 (females = 80)	MS	↑ (active) in females from the weaker side	↑ (latency) in males from the weaker side				↑ in females from the weaker side	↑ in males from the weaker side	
Pennisi et al., 2014	20 patients 20 controls	33.0 (24.0–45.0) 29.5 (26.0–45.5)	CD	=	=		↓	↑	↓		
Bella et al., 2015	13 patients	39.0 (24.0–46.0)	CD	↓			↓	↑	↓		
Pennisi et al., 2017	20 gluten-free 20 *de novo* 20 controls	35.1 ± 6.0 35.0 ± 12.0 33.4 ± 8.2	CD	=			↓	↑	↓ in *de novo*		
Tokimura et al., 2020	1 patient	72.0	GFAP		↑						
Fisicaro et al., 2021b	15 patients 15 controls	34.1 ± 12.0 34.9 ± 9.2	CD	=	=				↓	↓	
Lanza et al., 2021	15 patients 15 controls	34.07 ± 12.03 33.80 ± 9.29	CD	=		=					
Ozata et al., 1996	20 hypothyroidism 19 hyperthyroidism 20 controls	34.1 ± 2.4 35.2 ± 2.1 34.4 ± 2.6	Thyroid disease		↑ ↑						
Rizzo et al., 2008	10 hypothyroidism 10 controls	53.0 ± 8.0 NR	Thyroid disease	↑ = after therapy			↓ = after therapy	=	↑ = after therapy		
Terranova et al., 2016	4 thyroid hormone resistance 10 hypothyroidism 10 controls	20.5 (8.0–32.0) 53.0 ± 8.0 NR	Thyroid disease	= =			↑ ↓		↓ ↑		
Fried et al., 2017	21 patients 15 controls	66.9 ± 1.8 63.9 ± 2.2	Type-2 diabetes	=			=	=	=		↓
Fried et al., 2019	17 patients 14 pre-diabetes 9 controls	64.5 ± 7.5 65.7 ± 10.7 54. 9 ± 7.1	Type-2 diabetes Pre-diabetes	=							↓ ↓
Meyer et al., 1991	9 patients	29.4 (19.0–40.0)	WD		↑ in 6 patients						
Nolano et al., 1997	12 chronic stable 9 recurrent 10 without HE 14 controls	43.0–70.0 56.0–70.0 43.0–69.0 40.0–73.0	HE	↑ ↑	↑ ↑				↓ ↓ ↓		
Córdoba et al., 2003	20 pre-transplant 15 post-transplant	53.0 ± 11.0 53.0 ± 11.0	Minimal HE	↑ =	↑ = at upper limbs only						
Battaglia et al., 2005	36 patients 10 controls	58.0 ± 6.7 55.0 ± 6.0	end-stage renal disease	=			↓, = after dialysis	=, ↑ after hemodialysis	=		
McDonald et al., 2010	16 patients 8 post-transplant 11 controls	62.0 (40.0–78.0) 64.0 (55.0–71.0) 59.0 (40.0–73.0)	PBC	↑ =			↓ =	= =			
Cerri et al., 2010	11 patients 11 controls	51.0 ± 8.0 (41.0–68.0) 47.0 ± 4.0 (41.0–56.0)	PBC								↓ post-exercise facilitation
Golaszewski et al., 2016	15 patients 15 controls	58.0 ± 4.0 56.0 ± 2.0	Minimal HE	=							↓
Nardone et al., 2016b	15 patients 15 controls	58.0 ± 7.1 58.8 ± 8.2	Minimal HE	=			↑	↓			
Dubbioso et al., 2016	15 neurological 38 non-neurological 15 controls	28.2 ± 12.1 24.5 ± 7.50 26.7 ± 9.1	WD	↑	= =		↓	=	↓		
Hassan et al., 2019	15 patients 15 controls	64.1 ± 10.5 60.3 ± 6.2	HE	=, ↓ cerebellar inhibition							
Groiss et al., 2019	11 HE1 10 HE2 10 controls	59.5 ± 3.3 63.3 ± 2.4 60.5 ± 2.0	HE	=			= ↓	= =			
Dubbioso et al., 2020	3 NBIA 7 WD	31; 41; 55 33.5 ± 11.3	NBIA WD	=		=	↓ WD	=	↑ ↓		
Stezin et al., 2019	18 patients	18.9 ± 8.69	WD	↓ pre/post therapy	=						
Wu and Chu, 1996	1 patient	NR	Vit. B12 deficiency		↑, = upper limbs after therapy						
Oishi and Mochizuki, 1999	12 frontal atrophy 12 without atrophy 12 controls	56.9 ± 7.1 58.2 ± 7.3 57.9 ± 6.6	Chronic alcoholism		↑ =						
Ravaglia et al., 2002	62 patients 31 controls	NR	Chronic alcoholism		↑						
Misra et al., 2003	17 patients	NR	Vit. B12 deficiency		↑ in 8 out of 12, improve after therapy						
Atanassova et al., 2004	1 patient	44	Vit. B12 deficiency		↑, = after therapy						
Di Lazzaro et al., 2004	1 patient 5 controls	50 61.6 ± 18.7	Chronic alcoholism	↑							
Nardone et al., 2006	1 patient	48	Marchiafava–Bignami syndrome	↑, = after therapy	=					absent, = after therapy	
Nardone et al., 2010b	13 AWS patients 12 patients 15 controls	48.4 (26.0–68.0) 47.6 (25.0–66.0) 46.8 (24.0–65.0)	Alcohol withdrawal syndrome	= =	= =		= =	↑, = after riluzole =	= =		
Nardone et al., 2010a	8 patients 10 controls	54.4 (35.0–62.0) 53.0 (33.0–65.0)	Wernicke–Korsakoff syndrome	=	=	↓	=	=			
Misra and Kalita, 1998	9 patients	2.0–70.0	Herpes simplex encephalitis		=, except for one						
Kalita and Misra, 2002	65 patients	2.0–65.0	Japanese encephalitis		↑ or absent in 34						
Lo and Ratnagopal, 2007	1 patient	36.0	Bickerstaff’s brainstem encephalitis		↑, also cortico-bulbar						
Volz et al., 2016	34 patients 27 controls	28.0 ± 11.0 28.5 ± 10.0	NMDAR encephalitis	=			=	=	=		↓
Nardone et al., 2019b	1 patient 10 controls	56.0 NR	Rasmussen encephalitis	↓							
Ortelli et al., 2021	12 patients 12 controls	67.0 ± 9.6 64.3 ± 10.5	Post-COVID-19	=					↓ (post-pre		
Versace et al., 2021	12 patients 10 controls	67.0 ± 9.6 61.0 ± 8.2	Post-COVID-19	=		↓	↓ (also LICI)	=			
Nardone et al., 2012	10 patients 15 controls	64.8 ± 7.1 65.2 ± 6.8	RBD	=		↓	=	=			
Nardone et al., 2013	10 PD-RBD 13 PD 15 ctrls	65.9 ± 6.5 63.7 ± 6.4 66.4 ± 7.0	PD ± RBD	=		↓	=	=			
Das et al., 2013	13 patients 12 controls	47.7 ± 9.7 46.2 ± 10.5	OSAS	↑					↓		↓
Nardone et al., 2016a	13 patients 13 controls	46.2 ± 9.4 46.8 ± 5.4	OSAS	=		↓	=	=			
Rogić Vidaković et al., 2020	17 patients 12 controls	55.0 ± 12.0 46.0 ± 10.0	OSAS	↑		↓					
Restivo et al., 2000	18 patients	48.4 (26.0 ± 72.0)	SCA2	↑ (lower limbs)	↑ (lower limbs)						
Restivo et al., 2002	18 patients 20 controls	48.4 ± 14.7 44.4 ± 14.8	SCA2	↑ (lower limbs)			=	↓	↑		
Winner et al., 2004	2 patients	26; 31	SPG11							↓	
Huang et al., 2008	12 patients 12 controls	39.3 ± 8.1 35.7 ± 9.1	Sialidosis type I	=			↓	=	↓		
Mignarri et al., 2011	24 patiets	39.0 (18.0–57.0)	CTX		↑ (especially at lower limbs)						
Manganelli et al., 2014	3 patients	23; 26; 33	NPC			↓					
Benussi et al., 2017	4 patients (2 twins) 18 controls	25; 25; 34; 57 39.2 ± 15.7	NPC			↓, = after therapy	=	=			↓, = after therapy
Dubbioso et al., 2017a	2 patients	37; 37	Ch-Ac	=		↓	↓	↑	=		
Velázquez-Pérez et al., 2017	33 patients 33 controls	40.5 ± 10.8 (21.0–70.0) 43.6 ± 12.0 (19.0–71.0)	Pre-SCA2	↑ (at follow-up)	↑ (lower limbs, at follow-up)				=		

Each TMS parameter tested is indicated as increased (↑), reduced (↓), or not significantly modified (=) compared to healthy subjects or baseline condition. A non-significant difference does not demonstrate that a given parameter is unaffected by the disease, but it rather indicates that there is no evidence of change beyond the limits for which the study is powered. AWS, alcohol withdrawal syndrome; Ch-Ac, chorea-acanthocytosis; CD, celiac disease; CMCT, central motor conduction time; CTX, cerebrotendinous xanthomatosis; COVID-19, COronaVIrus disease-2019; cSP, contralateral cortical silent period; GFAP, glial fibrillary acidic protein astrocytopathy; HE, hepatic encephalopathy, grade 1 (HE1), grade 2 (HE2); iSP, ipsilateral silent period; MS, multiple sclerosis; ICF, intracortical facilitation; LICI, long-interval intracortical inhibition; LTP, long-term potentiation; NBIA, neurodegeneration with brain iron accumulation; NPC, Niemann-Pick disease type C; N, number of subjects; NMDA, N-methyl-D-aspartate receptor; NR, not reported; OSAS, obstructive sleep apnea syndrome; PBC, primary biliary cirrhosis; PD, Parkinson’s disease; RBD, REM-sleep behavior disorder; rMT, resting motor threshold; SAI, short-latency afferent inhibition; SCA2, spinocerebellar ataxia type 2; SICI, short-interval intracortical inhibition; SPG11, Hereditary spastic paraplegia with thin corpus callosum; WD, Wilson’s disease.

**TABLE 2:** Relevant repetitive transcranial magnetic stimulation (rTMS) studies in non-vascular secondary dementia or cognitive disorders.

References	N.	Age (years)	Disease	Repetitive TMS protocol	Main findings
Conte et al., 2008	13 patients 10 controls	49.0 ± 6.0 50.0 ± 4.0	Chronic ethanol abuse	5 Hz-rTMS over the left M1 (10 stimuli of 120% rMT) before and about 30 min after the intake of ethanol	Acute and chronic ethanol intake alters cortical excitability and short-term plasticity of the primary motor cortex
Qiao et al., 2016	18 test group 20 control group	49.0 48.0	Detoxified alcohol-dependence	10 Hz TMS over the right DLPFC, 20 sessions	Improvement in memory, which correlated with hippocampal brain metabolites detected by proton magnetic resonance spectroscopy
Jansen et al., 2019	39 patients 36 controls	41.6 ± 8.63 43.8 ± 10.9	Alcohol use disorder	Sham-controlled 10 Hz neuronavigated rTMS over the right DLPFC	rTMS can reduce the emotional impact of images as reflected in blood oxygenation level-dependent response in patients
Hulst et al., 2017	17 patients 11 controls	43.3 42.3	Multiple Sclerosis	Real 10-Hz over the right DLPFC, 110% rMT, 60 trains of 5 s, 25 s between trains (total 3,000 biphasic pulses in 30 min)	Improved working memory performance; the functional connectivity between the right DLPFC and the right caudate nucleus and bilateral cingulate gyrus increased
Manto et al., 2011	3 patients 3 controls	NR	NMDA-R encephalitis	Trains of high-frequency stimulation over the premotor area	Immunoglobulin G of patients markedly enhance the excitability of the motor cortex
Luber et al., 2008	15 SD healthy volunteers	24.5 ± 2.7	Cognitive impairment due to SD	Real 5 Hz over the left UMO, midline inferior parietal cortex, left LMO, 100% rMT, 7 s per trial, 35 stimuli per trial. Sham applied at the beginning and end of the session	Real rTMS only, delivered to UMO but not to LMO improved working memory No effect when the same participants were not sleep deprived
Luber et al., 2013	27 SD (13 rTMS vs. 14 sham) + 21 non-SD (10 active vs. 11 sham) healthy	23.3 ± 1.2 (active) 23.3 ± 0.6 (sham)	Cognitive impairment due to SD	5 Hz over the left UMO, 100% rMT, 7 s per trial, 35 stimuli per trial. Four 1.5-h sessions performed over the course of a 2-day SD period	Real rTMS group did not show slowing or lapsing and performed similarly to non-SD subjects. The SD group showed improvement of working memory 18 h after the last rTMS session
Martinez-Cancino et al., 2016	6 SD healthy volunteers	25.00 ± 2.61	Cognitive impairment due to SD	5 Hz over the left MFG (group 1) and left MOG (group 2), 100% rMT, single 20 min session (10 s stimulation, 50 s pause)	High-frequency rTMS in different brain targets determined improvement of working memory due to SD
Guo et al., 2019	17 SD healthy volunteers	23.00 ± 1.37 (21–26)	Cognitive impairment due to SD	5 Hz over the left DLPFC, 100% rMT, 20 trains of 50 pulses, 50-s intertrain interval, one session (total 1,000 pulses). Resting state functional magnetic resonance imaging and working memory test performed during a rested waking period, after SD and rTMS	high-frequency rTMS applied over left DLPFC may contribute to the recovery of the impaired working memory after SD by modulating the neural activity of related brain regions
Li et al., 2021	33 real group 33 sham group	25.8 ± 2.4 25.6 ± 2.4	Cognitive impairment due to SD	2 sessions of 10 Hz rTMS (40 trains of 50 pulses, 20-second intertrain interval) over the left DLPFC	Positive effects on hypothalamic-pituitary-adrenal axis and frontal activation, alleviating cognitive impairment

CSP, cortical silent period; DLPFC, dorsolateral prefrontal cortex; LMO, lower part middle occipital gyrus; M1, primary motor area; MEPs, motor evoked potentials; MFG, medial frontal gyrus; MOG, medial occipital gyrus; NMDA-R, N-methyl-D-aspartate receptor; NR, not reported; rMT, resting motor threshold; SD, sleep deprivation; UMO, upper part middle occipital gyrus.

The TMS group included 4 studies on NPH (Zaaroor et al., 1997; Chistyakov et al., 2012; Nardone et al., 2019a; Agrawal et al., 2021), 11 on MS (Cucurachi et al., 2008; Mori et al., 2011, 2013; Canham et al., 2015; Hofstadt-van Oy et al., 2015; Nantes et al., 2016; Chalah et al., 2018, 2019; Chaves et al., 2019, 2021a,b), 6 on CD and other immune-mediated disorders (Pennisi et al., 2014; Bella et al., 2015; Pennisi et al., 2017; Tokimura et al., 2020; Fisicaro et al., 2021b; Lanza et al., 2021), 5 on endocrine disorders (Ozata et al., 1996; Rizzo et al., 2008; Terranova et al., 2016; Fried et al., 2017, 2019), 13 on liver and renal diseases (Meyer et al., 1991; Nolano et al., 1997; Córdoba et al., 2003; Battaglia et al., 2005; Cerri et al., 2010; McDonald et al., 2010; Dubbioso et al., 2016; Golaszewski et al., 2016; Nardone et al., 2016b; Groiss et al., 2019; Hassan et al., 2019; Stezin et al., 2019; Dubbioso et al., 2020), 9 on nutritional and alcohol-related disorders (Wu and Chu, 1996; Oishi and Mochizuki, 1999; Ravaglia et al., 2002; Misra et al., 2003; Atanassova et al., 2004; Di Lazzaro et al., 2004; Nardone et al., 2006, 2010b, 2016a), 7 on infectious or inflammatory diseases (Misra and Kalita, 1998; Kalita and Misra, 2002; Lo and Ratnagopal, 2007; Volz et al., 2016; Nardone et al., 2019b; Ortelli et al., 2021; Versace et al., 2021), 5 on sleep disorders (Nardone et al., 2012, 2013, 2016a; Das et al., 2013; Rogić Vidaković et al., 2020), and 9 on rare genetic diseases (Restivo et al., 2000, 2002; Winner et al., 2004; Huang et al., 2008; Mignarri et al., 2011; Manganelli et al., 2014; Benussi et al., 2017; Dubbioso et al., 2017a; Velázquez-Pérez et al., 2018). The other group consisted of 10 articles applying rTMS in different clinical contexts (Conte et al., 2008; Luber et al., 2008, 2013; Manto et al., 2011; Martinez-Cancino et al., 2016; Qiao et al., 2016; Hulst et al., 2017; Guo et al., 2019; Jansen et al., 2019; Li et al., 2021).

**FIGURE 1:**
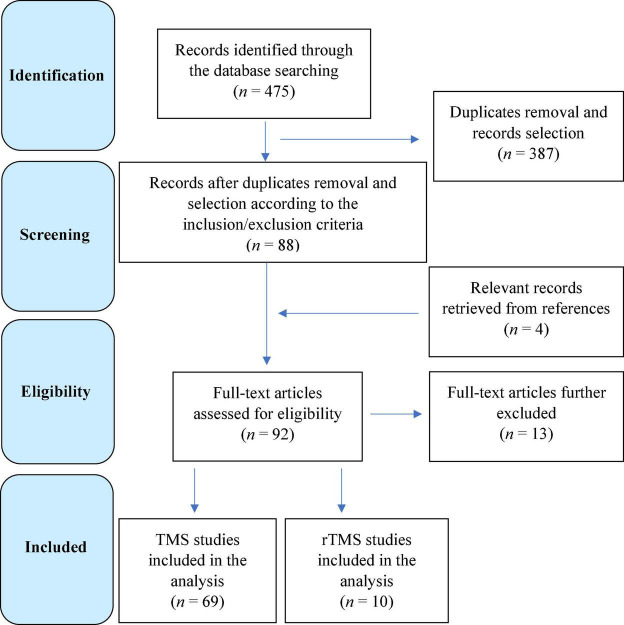
Flow diagram showing the search strategy, the number of records identified, and the number of included/excluded transcranial magnetic stimulation (TMS) and repetitive TMS studies (Moher et al., 2009).

Figure 1 contains the flow diagram showing the search strategy, the number of records identified, the numbers of excluded studies, and the number of those eventually included (Moher et al., 2009).

### Normal-pressure hydrocephalus

Idiopathic NPH, one of the main causes of secondary dementia, is characterized by the clinical triad of gait disturbance, cognitive dysfunction, and urinary incontinence. When NPH is promptly diagnosed, surgical ventricular shunt allows a rapid and substantial improvement of walking and cognitive status, till a complete resolution in some cases (Wang et al., 2020). For this reason, methods providing objective evidence of developing NPH are essential for differential diagnoses, selection criteria, and surgical decisions.

The first TMS study in patients with NPH (Zaaroor et al., 1997) demonstrated that the gait disturbance is not related to an impairment of conduction along the cortico-spinal tract, whereas another study (Chistyakov et al., 2012) showed a M1 disinhibition in terms of a significant decrease of rMT and SICI. The very low rMT from the leg motor area was the first and most prominent finding in the early stage of disease, whereas the decrease of rMT and SICI was not significant from the hand muscles (Nardone et al., 2019a). In the latter study, the significant reduction of SAI may indicate an impairment of cholinergic circuits and suggest that some patients with apparent idiopathic NPH may develop AD during the disease course. Overall, these data, although limited, indicate that NPH can determine a hyperexcitability of the motor cortex, mostly affecting the leg cortical representation in the early phases and a more widespread cortical pathology in the later stage. Such a pattern of electrocortical changes might be considered as a neurophysiological marker of NPH and a useful hint in differential diagnosis. Finally, a very recent study has evaluated the effect of lumbar drainage on cortical excitability in 11 NPH patients compared to 13 healthy controls (Agrawal et al., 2021). The results demonstrated a significant reduction in the lower limb rMT after lumbar drainage, along with a trend toward improvement in SICI and a shortening of the CMCT at lower limb. As a whole, these results seem to suggest a temporary increase in cortical excitability after lumbar drainage for NPH (Agrawal et al., 2021).

Taken together, TMS can guide the selection of those patients who will benefit from shunting, thus providing prognostic information. Moreover, from a pathophysiological perspective, these results support the view that an altered control of motor output, rather than the pure impairment of central motor conduction, is responsible for gait disturbances in NPH and that recovery of cortico-spinal excitability following ventricular shunt placement can correlate with clinical improvement.

Interestingly, the impairment of cholinergic neurons that can contribute to cognitive decline and gait impairment may also indicate the occurrence of AD pathology in some patients with NPH. Based on the observation that some cases of NPH developed AD during their course (Espay et al., 2017) and the poorer outcome than would be expected in patients with NPH alone (Pomeraniec et al., 2016), recent studies suggest the overlapping AD pathology in about 30% of NPH cases. Indeed, both NPH and AD build-up of brain metabolic wastes and amyloid-β plaques, perivascular reactive astrogliosis, and mislocalization of astrocyte aquaporin-4, thus suggesting that amyloid deposition might cause cholinergic dysfunction also in NPH (Graff-Radford and Jones, 2019). Moreover, in both AD and NPH, human studies have recently implicated an impaired glia-lymphatic (glymphatic) system, whose function facilitates brain fluid clearance and waste removal *via* the glia-supported perivascular channels (Reeves et al., 2020).

### Multiple sclerosis

Given that neurophysiological changes occur early in MS, TMS parameters may represent biomarkers of disease progression and response to drugs or rehabilitative interventions. To date, however, these changes have been only weakly associated with cognitive impairment (Simpson and Macdonell, 2015). A previous study (Cucurachi et al., 2008) found that a reduction in SAI was significantly related to cognitive dysfunction in MS. The study also showed that SAI improved after rivastigmine, as observed in AD. A recent review suggested a link between reduced acetylcholine expression, neuroinflammation, and cognitive deficits in MS (Polachini et al., 2018), although more evidence is required. Another study on 28 primary progressive MS patients found that evoked potentials, including MEPs, generally show a moderate correlation with clinical and functional status, but there was no association with any index of cognitive impairment (Canham et al., 2015).

A considerable amount of TMS investigations in MS showed significant abnormalities, especially regarding SICI and ICF, however, it is still unclear whether they could represent biomarker of disease progression, compensatory mechanisms, or treatment-related changes (Ayache et al., 2014, 2016; Ayache and Chalah, 2017). Moreover, longer cSP duration (Nantes et al., 2016) or triple stimulation technique (Hofstadt-van Oy et al., 2015) did not correlate with cognitive performance in relapsing-remitting MS. However, central mechanisms of fatigue, that can, at least in part, play a role in cognitive performances, appeared related to changes in cortical excitability, especially increased interhemispheric inhibition (Chalah et al., 2018) or SICI (Chalah et al., 2019), as well as attenuation of premotor-motor functional connectivity (Ruiu et al., 2020).

A recent TMS investigation aimed at identifying novel markers predictive of objective and subjective clinical features in 82 subjects, mostly affected by relapsing-remitting MS (Chaves et al., 2019). By means of a ratio considering the stronger and weaker side, a correlation between symptom severity and disability of MS and asymmetric brain excitability was found. Namely, subjects were less cognitively and physically impaired whether showing a cortical-spinal excitability asymmetry, being the weaker side more excitable than the stronger. The authors argued that the asymmetry might be a consequence of the hyperexcitability state secondary to a more prominent neuroinflammation in early MS impairing the weaker side (Chaves et al., 2019). Conversely, less inflammatory and more degenerative component might justify the shift of this excitability asymmetry in the later stages of the disease (Dutta and Trapp, 2014).

Very recently, a detailed single-pulse TMS protocol has been applied in a large cohort of MS patients with the aim to establish and validate a core-set of TMS variables that could predict typical clinical outcomes, including cognitive processing speed (Chaves et al., 2021b). Delayed and longer iSP, longer cSP, and higher rMT (all indicating a lower intercortical, intracortical, and cortico-spinal excitability) were found, especially in the hemisphere corresponding to the weaker hand. Moreover, greater interhemispheric asymmetry correlated with poorer performance in most of clinical outcomes (assessed by the Walking Speed, the nine-hole peg test from both the stronger hand and the weaker hand, fatigue, and symbol digit modality test), although TMS metrics were more related to motor than non-motor outcome measures (Chaves et al., 2021b).

Finally, two studies have assessed brain plasticity in MS with cognitive impairment using the intermittent theta-burst stimulation (iTBS) protocol. In the first study (Mori et al., 2011), altered cortical plasticity was observed in cognitively impaired patients, along with a direct correlation between liquoral content of amyloid-ß1–42 and the amount of LTP-like effect produced by iTBS. This indicates that neuroinflammation in MS might alter the metabolism of amyloid-ß with a reduction of its liquoral amount, thus producing defective cognitive functioning and maladaptive plasticity (Mori et al., 2011). In the second study, the authors probed if early started interferon (IFN) ß-1a (which is able to decrease the inflammatory component) at high dosage improved cognition and cortical functioning in 80 drug-free relapsing-remitting MS patients (Mori et al., 2013). Before treatment, those with gadolinium-enhancing (Gd +) lesions on brain magnetic resonance imaging (MRI) scored worse on cognitive tests and exhibited less pronounced plasticity after iTBS when compared with Gd-negative (Gd-) lesion individuals. After 6 months of treatment, both iTBS-related plasticity and cognition ameliorated in Gd + patients and were stable in those Gd-, thus suggesting that maladaptive plasticity and cognitive deficits might recover after IFN ß-1a at high dosage in *de novo* relapsing-remitting MS patients with Gd + lesions (Mori et al., 2013).

Based on the above-mentioned findings, although MEPs have demonstrated a good detection of disease progression and a reliable correlation with clinical disability, they have not been used as a measure of efficacy in clinical trials testing neuroprotective agents (Fernández, 2021). As such, TMS procedures need to be standardized and data interpretation made comparable between centers. For instance, introducing an automated and objective definition of MEP morphology could help in overcoming some of the barriers that currently limit a broader adoption of TMS (Yperman et al., 2020a). Another example could be the use of machine learning methods for MEP analysis, this approach showed predictive performance, although larger multi-center datasets are required in decision-making process and in the design of new treatments (Yperman et al., 2020b).

Very recently, however, it has been shown that a sex-specific disruption in cortico-spinal excitability may exist in MS (Chaves et al., 2021a): namely, males were more likely to have cognitive impairment as measured by the Montreal Cognitive Assessment (MoCA), whereas greater TMS asymmetry was noted in females, who demonstrated higher active MS and longer cSP in the hemisphere corresponding to the weaker hand. Males, but not females, exhibited asymmetry of nerve conduction latency (delayed MEP latency in the hemisphere corresponding to the weaker hand) and a relationship between delayed onset of iSP (measured in the hemisphere corresponding to the weaker hand) and MoCA, thus suggesting a cross-callosal disruption (Chaves et al., 2021a).

### Celiac disease and other immune-mediated disorders

Adult patients with CD can present several neurological and neuropsychiatric symptoms, including cognitive complaints, which may range from a mild “brain fog” to an overt dementia. The few TMS studies available converge on a “hyperexcitable celiac brain” (reduced SICI and cSP, increased ICF), which partially recovers when a sustained period of gluten-free diet (GFD) is adopted (Pennisi et al., 2014, 2017; Bella et al., 2015). As such, since GFD may exert a neuroprotective role, it needs to be adopted very early, though its effects on neurological manifestations (and particularly on cognitive features) is debated yet (Lanza et al., 2018a). More recently, the excitability of transcallosal interhemispheric connections, assessed by the latency and duration of iSP, was explored in GFD-naïve CD subjects with respect to healthy individuals (Fisicaro et al., 2021b). The iSP resulted to be significantly shorter in CD and a direct correlation between iSP duration and cognitive score was found, compatible with an interhemispheric disinhibition and adding support to the impairment of GABA-mediated cortical and callosal circuitries (Fisicaro et al., 2021b). Very recently, central cholinergic functioning explored by SAI of the motor cortex resulted to be not affected in *de novo* CD patients compared to age-matched healthy controls, although the patients presented a statistically significant worse score at MoCA (Lanza et al., 2021). This finding might add support to the vascular inflammation hypothesis of the “gluten encephalopathy,” which seems to be due to an etiology different from that of the cholinergic dysfunction (Lebwohl et al., 2016).

Abnormal evoked potentials, including prolongation of MEP conduction times, have been recently reported in a 72-year-old female developing progressive impairment of cognition and gait, eventually leading to the diagnosis of autoimmune glial fibrillary acidic protein astrocytopathy (GFAP) based on the typical MRI findings of periventricular radial linear Gd enhancement and longitudinally extensive lesions in the spinal cord, and the anti-GFAP antibody detected in the cerebrospinal fluid (Tokimura et al., 2020).

No TMS study was performed in other immunologically mediated disorders, including primary or secondary vasculitis with central nervous system (CNS) involvement (Mansueto et al., 2022).

### Endocrine disorders

Prolonged CMCT was reported in patients with both hypo- and hyperthyroidism, although no correlation was found with levels of free thyroxine (FT4), free 3, 3′, 5-L-triiodothyronine (FT3), and thyroid stimulating hormone (TSH), as well as with onset age and disease severity or duration. Notably, TMS changes did not entirely improve after restoration of euthyroidism (Ozata et al., 1996). A more extensive cortical excitability study was carried out in 10 patients with overt hypothyroidism and 10 age-matched healthy controls (Rizzo et al., 2008). At baseline, patients showed decreased cortical excitability, with increased rMT and decreased steepness of MEP recruitment curves. These changes were paralleled by longer cSP and decreased SICI. After 3 months of replacement therapy, all parameters restored to normal values, except for SICI, which needed 6 months of therapy to normalize (Rizzo et al., 2008). A more recent study has confirmed that thyroid hormones influence cortical inhibitory circuits. Accordingly, in overt hypothyroidism, cSP was prolonged and SICI decreased, whereas those with thyroid hormone resistance (elevated level of thyroid hormones but unsuppressed TSH) showed the opposite (Terranova et al., 2016). In patients with hypothyroidism, an impaired SICI [which is a measure of GABA-A receptor-mediated activity (Ziemann et al., 2015; Cash et al., 2017)] supports the hypothesis that thyroid hormones not only modulate cortical excitability but they can also influence the cortical inhibitory circuits.

As known, type-2 diabetes mellitus (T2DM) accelerates cognitive aging and increases risk of dementia. A previous prospective cross-sectional study (Fried et al., 2017) on 21 adults with T2DM and 15 demographically similar non-diabetic controls explored M1 LTP-like plasticity by comparing the MEP amplitude before and after iTBS. An abnormal cortico-motor plasticity that correlated with reduced verbal learning was found in patients. Moreover, since both iTBS after-effects and the Rey Auditory Verbal Learning Task performance are both NMDA receptor-dependent measures, their relationship in T2DM may reflect an impaired glutamatergic neurotransmission (Fried et al., 2017). In a subsequent study combining TMS with magnetic resonance spectroscopy (MRS) (Fried et al., 2019), neuroplastic mechanisms were found to be impaired also in pre-diabetes, whereas in T2DM patients reduced cortico-motor plasticity was associated with lower cortical glutamatergic metabolite concentration. These findings suggest that glutamate-mediated neurotransmission might represent a therapeutic target (Fried et al., 2019).

To our knowledge, no study is available in cognitive disorders due to other endocrine diseases.

### Hepatic and renal diseases

Motor evoked potentials in hepatic encephalopathy (HE) are able to detect and monitor alterations in neurotransmission, likely related to myelin changes and microadenoma (Amodio and Montagnese, 2015). A single study reported a significant increase in rMT and CMCT, a reduction of the MEP/compound muscle action potential amplitude ratio, and a shorter cSP, thus suggesting that the inhibitory interneuronal network dysfunction plays a role in HE patients (Nolano et al., 1997).

Given that a previous experimental study reported that hyperammonemia leads to opposite changes of GABAergic neurotransmission with an increase in the cerebellum and a decrease in the cerebral cortex, the cerebellum GABA-mediated activity has been examined in 15 HE subjects with different disease severity (Hassan et al., 2019). The authors utilized a different form of paired-pulse TMS, i.e., the cerebellar inhibition (CBI), which is based on the evidence that a conditioning magnetic stimulus on the cerebellum reduces the MEP amplitude elicited by the test stimulus on contralateral M1 at 5–7 ms interstimulus intervals. CBI is considered to be generated by the activity of cerebellar Purkinje cells and subsequent GABA-mediated suppression of the dentato-thalamo-cortical tract (Ugawa et al., 1991; Groiss and Ugawa, 2012). This paradigm, applied in subjects with disorders affecting the cerebellum, such as spino-cerebellar ataxias, late onset ataxias, acquired cerebellar diseases, and multiple system atrophy, has revealed decreased CBI secondary to a damage in the cerebello-thalamo-cortical tract or in the cerebellum itself (Ugawa et al., 1994a,b, 1997; Grimaldi et al., 2014). HE patients showed a decreased cerebellar inhibition (in terms of increased GABA-mediated activity of Purkinje cells) that was related to disease severity, suggesting a dysfunction of the efferent cerebellar pathways (Hassan et al., 2019). A recent investigation supports this result showing a reduction of GABArgic inhibition, correlated with HE severity, although this was associated with an overall reduced cortico-spinal excitability (Groiss et al., 2019).

Of note, in minimal HE (i.e., the earliest form of HE presenting with psychomotor slowing and subtle impairment of cognition), paired-pulse TMS showed a significant increase of SICI and a reduction of ICF, suggesting an imbalance between excitatory and inhibitory intracortical mechanisms toward an increased inhibitory transmission (Nardone et al., 2016b). Furthermore, associative sensory-motor index of plasticity, an indirect measure of motor learning assessed by PAS protocol, resulted to be impaired in minimal HE patients with respect to age-matched healthy controls (Golaszewski et al., 2016). Finally, T2-MRI hyperintensity observed along the cortico-spinal tract in cirrhotic patients were found to relate to functional abnormalities (including increased rMT, decreased MEP amplitude, and prolonged CMCT, all more marked in the lower extremities) and to revert (except for CMCT at lower limbs) after liver transplantation. These findings suggest that mild cerebral edema along the cortico-spinal pathway may cause a neuronal dysfunction, clinically manifesting in minimal HE (Córdoba et al., 2003).

Wilson disease (WD) is a potentially treatable, inherited metabolism disorder characterized by the pathological accumulation of copper. Although the clinical course can vary, a progressive liver disease is a common feature, as well as some neurological (including cognitive) and psychiatric symptoms. With early diagnosis and treatment, the prognosis is favorable; however, it is of pivotal importance to diagnose patients before the onset of serious symptoms, such those related to CNS dysfunction (Członkowska et al., 2018). A systematic review of TMS in WD concluded for abnormal MEP features in 20–70% of participants, whereas motor responses could not be recorded in 7.6–66.7% of patients. Four studies reported significantly increased cortical excitability and prolonged CMCT, whereas one study found absent or prolonged MEP latency in 2/3 of patients (Bembenek et al., 2015). Interestingly, early diagnosis and prompt therapy of WD can prevent the occurrence of neurological signs, even at subclinical levels, as demonstrated by the normality of brain MRI, clinical scales, cognitive tests, and TMS measures of cortical excitability, including rMT, SICI, and cSP (Dubbioso et al., 2016). However, a previous study on 9 patients (6 of them with prolonged CMCT, reduced MEP amplitude, or absent responses in at least one of the examined muscles) revealed that in one patient only the abnormal responses normalized following treatment with penicillamine (Meyer et al., 1991). A reduction of cortical excitability, characterized by shortening of cSP and increasing of SICI and rMT, occurs when neurological signs become manifest (Dubbioso et al., 2016, 2020). Another recent study has described the longitudinal changes of rMT and CMCT in 18 WD patients. A progressive decrease of rMT was observed, whereas CMCT did not improve despite chelation therapy, thus suggesting that rMT may serve as a marker of chelation efficacy (Stezin et al., 2019).

Primary biliary cirrhosis (PBC) is a female-predominant autoimmune disease associated with fatigue, memory impairment, and sleep disturbances. It has been shown that PBC patients have impaired central activation and abnormal SICI (suggesting CNS abnormalities beyond the voluntary control) and that ICI and SICF were related to daytime somnolence (McDonald et al., 2010). However, both transplanted and non-transplanted patients showed similar abnormalities, raising questions about the mechanisms underpinning these changes and the neurological dysfunction in PBC (McDonald et al., 2010). Finally, compared to healthy controls, female patients did not show post-exercise depression of cortical excitability after voluntary submaximal tonic contraction of finger flexor muscles until exhaustion (Cerri et al., 2010). This might indicate an impairment of the neural mechanisms underlying central fatigue that occur in some PBC patients (Cerri et al., 2010).

Changes of cortical excitability have been also reported in the end-stage renal disease, with the recovery of SICI after hemodialysis, peritoneal dialysis, and transplantation (Battaglia et al., 2005).

### Nutritional and alcoholic-related disorders

Central motor conduction time was found to be prolonged in cognitive decline due to vitamin B_12_ deficiency (Atanassova et al., 2004), although its prolongation might be related to a degeneration/demyelination of the lateral spinal columns. Another study showed that MEPs and MRI changes were consistent with myelin alteration and that the outcome at 6 months correlated with MEP changes (Misra et al., 2003). Given that the central motor pathway may respond to hydroxocobalamin therapy, MEPs can be useful in the monitoring of functional recovery following replacement treatment (Wu and Chu, 1996). To date, no study has been carried out in cognitive disorder due to other vitamin deficiency.

Five studies investigated the central motor conductivity in alcohol-related cognitive disorders: CMCT was significantly prolonged in the chronic alcoholics with frontal lobe atrophy, with a significant positive correlation between CMCT and atrophy severity (Oishi and Mochizuki, 1999). Additionally, in chronic alcoholism, the lack of TMS-evoked I waves pointed toward an impairment of intracortical neural circuitry (Di Lazzaro et al., 2004), whereas a significant CMCT prolongation correlated with frontal skills impairment on neuropsychological testing (Ravaglia et al., 2002). In one study comparing patients with the alcohol withdrawal syndrome, patients with chronic alcohol abuse, and healthy subjects, ICF was increased in the first group only, although it could be restored after administration of the glutamate receptor antagonist riluzole. Other parameters, such as rMT, SICI, and cSP, were not affected, supporting the hypothesis of a selective glutamatergic dysfunction during alcohol withdrawal (Nardone et al., 2010b).

In Marchiafava-Bignami’s syndrome, iSP abnormalities have been reported, along with an extensive abnormal signal intensity throughout the entire corpus callosum at MRI, thus suggesting a severe callosal demyelination (Nardone et al., 2006). Finally, a reduction of SAI was described in patients with amnesia due to Wernicke-Korsakoff syndrome (WKS), thus demonstrating the impairment of cholinergic cortical networks involved in SAI. However, none of the correlations between SAI and neuropsychological tests reached a statistical significance (Nardone et al., 2010a), suggesting that cholinergic dysfunction could not account for the memory disorder in these patients and that damage to the cholinergic system alone was not sufficient to cause a persisting amnesic disorder. It is likely that multiple neurochemical changes, including interactions between cholinergic and non-cholinergic neuromodulatory systems, underlie this amnesic syndrome (Nardone et al., 2010a). Accordingly, recent “pharmaco-TMS” evidence reveals that SAI can be influenced by other neurotransmitters (such as GABA) in addition to acetylcholine (Turco et al., 2018), and this should be considered when interpreting the lack of correlation between SAI values and neuropsychological scores observed by Nardone and colleagues (Nardone et al., 2010a).

### Infectious and inflammatory diseases

A previous review in Herpes simplex encephalitis concluded that changes in evoked potential, including MEPs, are infrequent and, if present, an association with HIV infection should be suspected (Misra and Kalita, 1998). No study is available in other infectious diseases of the CNS.

Cortical excitability after PAS was found to be decreased in anti-NMDA receptor encephalitis (Volz et al., 2016). Also, the lower PAS-induced plasticity significantly correlated with the modified Rankin Scale and with a lower functional connectivity within the motor network. Follow-up assessments, available in 6 patients, demonstrated a parallel improvement of PAS-induced plasticity and functional status. These findings indicate that neurophysiological mechanisms, including cortical excitability, LTP-like plasticity, GABAergic and glutamatergic functioning, are altered in patients with anti-NMDA receptor encephalitis and they can be helpful in diagnostic, prognostic, and response to therapy (Volz et al., 2016).

Bickerstaff’s brainstem encephalitis (BBE) is a rare post-infectious neurological disease characterized by external ophthalmoplegia, ataxia, lower limb areflexia, extensor plantar response, and disturbance of consciousness (drowsiness and stupor, till coma). Cortico-hypoglossal and cortico-spinal conduction abnormalities were also reported in BBE, thus further supporting the multifaceted manifestations of anti-GQ1b IgG antibody-positive spectrum disorders (Lo and Ratnagopal, 2007). Finally, compared to other evoked potentials, MEP changes occurred more frequently in Japanese encephalitis, also with a prognostic value (Kalita and Misra, 2002).

Recently, the motor output has been probed in a 56-year-old man affected by acquired unilateral hemispheric atrophy secondary to Rasmussen encephalitis (Nardone et al., 2019b). Namely, the amplitude of the ipsilateral MEPs was higher than those of ten age-matched healthy individuals, thus demonstrating a reinforcement of motor ipsilateral projections from motor cortex unaffected to the affected limb (Nardone et al., 2019b).

Lastly, a non-negligible amount of subjects complains of cognitive impairment, apathy, and fatigue after COronaVIrus Disease-2019 infection (COVID-19) (Fisicaro et al., 2021a). The evaluation of rMT, MEP amplitude, and cSP duration from the left M1, before and 2 min after a fatiguing isometric pinching task, pointed out an overall GABAergic impairment, possibly representing the correlate of fatigue and explaining apathy and executive deficits observed in post-COVID-19 patients (Ortelli et al., 2021). A subsequent study explored the activity of inhibitory intracortical circuits and sensory-motor interactions in patients complaining of fatigue and presenting executive dysfunction after resolution of COVID-19 with neurological complications (Versace et al., 2021). TMS studies revealed a marked reduction of SICI, a disruption of long-interval intracortical inhibition (reflecting GABA-B-mediated inhibition), and a decrease of SAI, thus suggesting a dysfunction of GABAergic and cholinergic activity in the M1 in patients who recovered from COVID-19 with cognitive manifestations (Versace et al., 2021).

### Sleep disorders

Transcranial magnetic stimulation changes have been reported in obstructive sleep apnea syndrome (OSAS), restless legs syndrome (RLS), insomnia, and sleep-deprived (SD) healthy subjects. Overall, OSAS tends to exhibit an increased motor cortex inhibition while the opposite is observed in RLS (Lanza et al., 2015), whereas excitability seems to be in favor of a profile of cortical disinhibition in chronic insomnia and SD individuals. Brain plasticity abnormalities have been demonstrated both in OSAS and RLS (Das et al., 2013; Lanza et al., 2018b,c, 2022b). SAI was significantly reduced in apneic patients compared to healthy controls, and SAI level was strongly correlated with neuropsychological scores, suggesting that cognitive deficits in OSAS may be, at least in part, related to impaired cholinergic neurotransmission, presumably caused by the intermitted nocturnal hypoxemia (Nardone et al., 2016a). A more recent navigated TMS study demonstrated an increased rMT and reduced SAI in OSAS, thus confirming previous findings of impaired cortical afferent inhibition in these patients (Rogić Vidaković et al., 2020).

In patients with idiopathic REM-sleep behavior disorder (RBD), which is a strong predictor of neurodegeneration (Mahowald and Schenck, 2018), mean SAI was found to be significantly reduced (Figorilli et al., 2021). SAI also correlated with episodic verbal memory and executive functions, supporting the hypothesis of cholinergic dysfunction and risk of cognitive impairment in some RBD patients (Nardone et al., 2012). SAI abnormalities were also revealed in RBD with a diagnosis of Parkinson’s disease (PD), thus suggesting that RBD-related cholinergic dysfunction may be a significant determinant of cognitive impairment in PD (Nardone et al., 2013; Fisicaro et al., 2020b). In RBD patients still asymptomatic for a parkinsonian syndrome, changes of ICF and, to a lesser extent, of SICI has been observed and these changes might precede neurodegeneration. Moreover, SICI correlated with muscle tone alteration, adding support the RBD model of retrograde influence on the cortex from the brainstem (Lanza et al., 2020a). Very recently, a direct TMS comparison between 15 patients with idiopathic RBD and 15 with PD with RBD, both age-matched to 15 healthy participants, showed that both patients’ groups shared a reduced ICF, thus suggesting the involvement of glutamatergic transmission not only in subjects at risk for degeneration but also in those with an overt α-synucleinopathy (Lanza et al., 2022a).

### Rare genetic diseases

Niemann-Pick disease type C (NPC), a rare autosomal recessive lysosomal storage disorder, presents with a wide range of neurological manifestations, including cognitive impairment (Iodice et al., 2015). In three patients affected by the adult form of NPC, an alteration of SAI reduction was found (Manganelli et al., 2014), thus suggesting that cholinergic changes might play a role in the development of cognitive impairment in NPC (Manganelli et al., 2014) and strengthening the similarity with AD (Esposito et al., 2019). Another recent TMS study on two patients carrying a homozygous mutation in the *NPC1* gene and in two heterozygous family members found that baseline SAI and LTP-like plasticity were impaired. After 12 months of treatment with miglustat, a considerable improvement of SAI and LTP-like plasticity was observed in the two patients, suggesting that these biomarkers might support the diagnosis and provide prognostic insights (Benussi et al., 2017). A further study showed that long term therapy with miglustat did not arrest the cognitive decline or improve the reduction of SAI, although it could stabilize other neurological manifestations (Tozza et al., 2018).

In chorea-acanthocytosis, an autosomal recessive neurodegenerative disorder characterized by adult-onset chorea, acanthocytes in the peripheral blood, and HD-like neuropsychiatric symptoms, a striking alteration of SICI was found, supporting the functional disruption of GABA-mediated networks (Dubbioso et al., 2017a). The authors also demonstrated a reduction of SAI, suggesting that cognitive and psychiatric disturbs might be underpinned by central cholinergic dysfunction (Dubbioso et al., 2017a).

A recent TMS study compared patients with neurodegeneration with brain iron accumulation (NBIA), such as patients with aceruloplasminemia and Kufor-Rakeb disease (*PARK-9*), and those with neurological WD. NBIA exhibited abnormal prolongation of cSP with respect to WD. On the contrary, WD displayed higher rMT and reduced cSP and SICI (Dubbioso et al., 2016, 2020). SAI, tested in NBIA only, was normal, suggesting a non-cholinergic origin of cognitive impairment in these subjects (Dubbioso et al., 2020).

In patients with spinocerebellar ataxias type 2 (SCA2), cortico-spinal tract involvement has been frequently reported (Restivo et al., 2000; Giardina et al., 2020), even in the pre-symptomatic stage (Velázquez-Pérez et al., 2017), and it seems to progress significantly during the pre-ataxic stage (Velázquez-Pérez et al., 2018). A first study (Restivo et al., 2002) found that mean cSP duration and rMT were significantly increased in the patient group. Conversely, SICI showed no significant difference between patients and controls, whereas at longer interstimulus intervals the expected facilitation of test responses resulted significantly less marked in patients. Notably, these alterations were related to the worsening of the general clinical status. The authors speculated that changes of motor cortex excitability in SCA2 might represent a slow neurodegenerative process characterized by a gradual loss of cerebellar neurons, eventually leading to an imbalance between inhibitory and excitatory circuits within the motor system (Restivo et al., 2002).

Similarly, TMS revealed cortico-spinal alterations even in subjects with mild clinical signs of cortico-spinal tract involvement in cerebrotendinous xanthomatosis (a rare neurometabolic disease due to defective activity of sterol 27-hydroxylase, with plasma and tissue cholestanol storage) (Mignarri et al., 2011), the clinical phenotype of which is characterized by both systemic manifestations and neurological signs, including cognitive decline. Namely, lower-limb CMCT was increased in 15 out of 24 (62.5%) patients, with interside difference abnormal in 9 (37.5%), whereas upper-limb CMCT was prolonged in 9 out of 24 (37.5%) with interside difference abnormal in 12 out of 24 (50%). MEPs at rest were absent in 13 out of 24 (54.2%) patients from lower limbs and in 4 out of 24 (16.7%) from upper limbs (Mignarri et al., 2011). Interestingly, MEPs revealed pyramidal tract alterations also in patients with subclinical signs only (i.e., pyramidal signs without disability), although no significant correlation was found between MEP alterations and Pyramidal Function Scale. Replacement treatment can prevent the progression of pyramidal damage, especially if started early (Mignarri et al., 2011).

A progressive axonal degeneration was proven to occur in the cortico-cortical projections (lack of transcallosal inhibition) and cortico-spinal tract (reduced MEP amplitude) in hereditary spastic paraplegia with thin corpus callosum (*SPG11* gene) in two sisters with cognitive impairment (Winner et al., 2004). Lastly, in sialidosis type I, a rare inherited neurodegenerative disorder caused by mutations in the *NEU1* gene, the slope of input/output was significantly increased, whereas SICI and cSP duration were reduced in patients compared to controls, suggesting that the major clinical changes take place above the brainstem (Huang et al., 2008).

### Therapeutic applications

To date only one study assessed the effects of high frequency-rTMS over the right dorsolateral prefrontal cortex (DLPFC) on working memory in a small sample of MS patients compared to healthy controls (Hulst et al., 2017). The authors reported that task accuracy improved after real but not sham (fictitious) stimulation. Moreover, the task-related functional connectivity between the right DLPFC and right caudate nucleus and bilateral cingulate gyrus increased, suggesting a rTMS-induced change in network efficiency in cognitively impaired MS subjects (Hulst et al., 2017).

Few studies were performed in cerebellar degeneration (Lanza et al., 2019b), reporting an improvement in cognitive performance attributed to an enhanced motor function and resource utilization. Cerebellar rTMS might also have enhanced prefrontal functions through the cerebellar projections into this area, which, in turn, improved cognitive abilities (Pope and Miall, 2014).

Anecdotally, patients with alcohol-related cognitive deficit showed no LTP-like effect after 5 Hz-rTMS over the left M1, while the cSP (which was already increased at baseline) remained unchanged (Conte et al., 2008). The positive effect of high-frequency rTMS over the right DLPFC on the cognitive regulation of emotional processing, reappraisal, and craving in alcohol use disorder has been recently observed in 39 patients and 36 healthy controls in an rTMS/functional MRI study, thus implying that rTMS can reduce the emotional impact of images, as reflected in blood oxygenation level-dependent response in these patients (Jansen et al., 2019). The positive effect of high-frequency rTMS over the same cortical target (right DLPFC) on memory was also noted and appeared to correlate with increased in brain metabolites detected by proton (MRS) in recently detoxified alcohol-dependent patients (Qiao et al., 2016).

At a short latency between a peripheral nerve (conditioning) stimulus and a second stimulus (called test stimulus) applied contralaterally on motor cortex, the corticomotor response is increased due to facilitation, a phenomenon called afferent facilitation of the motor response. Following high-frequency rTMS over the prefrontal areas of severe subacute NMDA receptor antibody encephalitis, the afferent facilitation of motor responses increased by immunoglobulin (Ig)-G administration, highlighting that IgG may induce hyperexcitability (Manto et al., 2011).

Finally, the effect of rTMS on cognitive impairment induced by SD has been assessed. A first study in 2008 demonstrated that 5 Hz-rTMS over the upper-middle occipital cortex resulted in a reduction of the sleep-induced reaction time deficit, without a corresponding decrease in accuracy (Luber et al., 2008). Few years later, the same research group showed the beneficial effects of rTMS after 4 sessions of concurrent rTMS/task performance given over the course of 2 days of SD (Luber et al., 2013). Similar results were reported by other authors, with 5 Hz-rTMS over the left middle occipital gyrus, resulting in greater accuracy and shorter response times after SD (Martinez-Cancino et al., 2016; Guo et al., 2019). Lastly, a recent study has evaluated the effect of high-frequency rTMS on reversing the negative effects of SD for 24 h in 66 healthy individuals randomized into “real” rTMS group and “sham” group (Li et al., 2021). The authors showed that SD induced cognitive impairment, increase of anxiety, depression, cortisol levels, and brain-derived neurotrophic factor (BDNF) levels, whereas decreased frontal blood activation. Notably, rTMS improved the hyperactivity of the hypothalamic-pituitary-adrenal axis, decreased the frontal blood activation induced by SD, and reduced the consumption of plasma proBDNF (Li et al., 2021).

## Discussion

### Summary of the evidences

A first consideration that can be drawn from the present review in that, while in degenerative dementia neurophysiological alterations might mirror specific, and possibly primary, neuropathological changes (and hence be used as early biomarkers), this pathogenic link appears to be overall weaker for secondary forms of dementia, in which neurotransmitter dysfunction is more likely related to a systemic or diffuse neural damage. In these cases, therefore, an effort toward the understanding of pathological mechanisms of cognitive impairment should be made, also by investigating the relationship between functional alterations of brain circuits and the specific mechanisms of neuronal damage triggered by the causative disease.

A common factor that seems to emerge is that the development of hyperexcitability/disinhibition of cortical circuits may be secondary to several noxious conditions, and, as such, it might represent a compensatory phenomenon that might prevent the development of an excitatory dysfunction; alternatively, it might be the consequence of a predominant impairment of inhibitory interneurons. This hypothesis contrasts with the view of hyperexcitability as a possible causative factor in some neurodegenerative disorders, such as AD and dementia in motor neuron diseases (Ranieri et al., 2020; Di Lazzaro et al., 2021). Interestingly, a hyperexcitability is also suggested in a few secondary dementias for which more homogeneous studies are available. Namely, in NPH, where hypoperfusion is hypothesized to underlie the onset of clinical manifestations, TMS showed a reduced GABAergic transmission and a hyperexcitability of the motor cortex (Chistyakov et al., 2012; Nardone et al., 2019a).

On the other hand, in MS neuroinflammation is the main noxious factor and it has been related to cortical hyperexcitability before the occurrence of structural alterations (Simpson and Macdonell, 2015; Chaves et al., 2019). In a recent large series (Chaves et al., 2021b), measures of intracortical inhibition (i.e., cSP and iSP) did not correlate with cognitive changes, while reduced cortico-spinal excitability (i.e., increased rMT and reduced MEP amplitude) correlated with both motor impairment and cognition, possibly indicating that the later stages of the disease are more affected by neurodegeneration and greater disability. Nevertheless, sex-specific disruption in cortico-spinal excitability and hemispheric asymmetry have been recently found in MS, pointing to interhemispheric disruption as a potential biomarker of cognitive impairment and a possible target for neuromodulation treatments (Chaves et al., 2021a).

Other intriguing considerations arise from the comparison of NPH and MS. As known, the most relevant pathophysiological mechanism underlying cognitive decline in MS implies the degeneration of cortical and subcortical networks. NPH may be also characterized by some neurodegenerative mechanisms, which, at least in part, seem to overlap with those observed in some primary dementia (including AD and FTD). Similarly, a common mechanism for neurodegeneration in MS and AD has been suggested, likely due to an increased amyloid precursor protein expression in the axons around MS plaques (Chandra, 2015). In this context, blood flow and pulsation propagation changes have been demonstrated in MS patients, which are similar to those of NPH patients, and consistent with an underlying pulse wave encephalopathy component in MS (Bateman et al., 2016). Moreover, although cytokine production and immune activation have been associated with various degenerative and neuroimmune disorders (such as MS), inflammatory changes have been reported also in NPH. The enlarged brain ventricles may indeed activate the production of interleukin (IL)-1β, IL-6, IL-10, IL-21, and tumor necrosis factor-α, with a pattern similar to that observed in MS, thus reflecting an inflammatory component in NPH as well (Sosvorova et al., 2015). Translationally, this might be viewed as an example of how improve the classification of secondary dementias according to the putative etiological contribution of neurodegeneration processes, although current classifications do not fully consider it, except for the possibility of “mixed dementia” (e.g., of vascular and degenerative origin).

Among immune-mediated disorders, CD might affect CNS through multiple pathogenic factors in addition to the cross-reacting antibodies and immune complex deposition, including direct neurotoxicity and, especially in severe cases or in non-GFD patients, nutrients deficiency secondary to malabsorption. In this case, the predominant neurophysiological pattern is a hyperexcitability characterized by reduced GABAergic inhibition and facilitated glutamatergic transmission. However, hyperexcitability does not seem to be similarly associated with hyperammonemia-related damage in HE, where inconsistent results have been reported about changes in SICI, with reduced inhibition possibly characterizing only the severe stages (Groiss et al., 2019). Finally, in other cognitive disorders associated with mechanisms involving specific molecular targets (such as the anti-NMDA receptor encephalitis), circuitry dysfunction appears more strictly related to these pathogenic factors, in which an altered LTP-like linked to NMDA receptor-dependent plasticity has been demonstrated (Volz et al., 2016).

Of note, SAI is known to be reduced in primary cholinergic forms of dementia, such as AD and DLB (Di Lazzaro et al., 2021). Few reports indicate a reduction of SAI also in some secondary dementias, thus leading to hypothesize that central cholinergic dysfunction may contribute to cognitive decline. Interestingly, some of these alterations can be reverted by the specific treatment targeting the neuropathological mechanisms underlying these disorders, e.g., surgical drainage in NPH, immunomodulatory therapy in MS and autoimmune encephalitis, or GFD in CD.

### Current limitations and research perspectives

Currently, no distinctive TMS pattern can be identified that is prompt to be used to predict the occurrence or progression of cognitive decline in a specific pathological condition. However, to date, most pathological hypotheses and proposed applications of TMS in secondary dementia remain speculative, mainly due to limiting factors in the collection and analysis of the articles reviewed, that prevent a meta-analysis approach. These mainly consist of the small sample size and heterogeneity of the pathological condition examined. Furthermore, most of the studies are based on inclusion criteria that are not specifically targeted on the presence and quantification of cognitive impairment. Additionally, due to the average small sample size of the individual studies, even in those conditions where a given parameter was consistently affected among different reports (such as the reduction of SICI in NPH and CD), the effect of other potential confounding factors, such as a “publication bias,” cannot be excluded. Therefore, unlike primary forms of dementia, such as AD, DLB, and FTD, where TMS indexes might be not far from a “bench-to-bedside” translation (Di Lazzaro et al., 2021), the heterogeneous and often limited data available in secondary forms of dementia do not allow to foresee a prompt diagnostic translation yet.

Additionally, as emerged from the neurological conditions here reviewed, it is still unclear which TMS abnormality can be related to the disease *per se* and which would be strictly related to dementia within the same disease. Nonetheless, specific TMS measures of cortical excitability were found to be consistently altered in some of the above-mentioned disorders, such as: rMT in NPH, HE, and OSAS; cSP in MS, CD, HE, and hypothyroidism; SAI in OSAS, RBD, and NPC; SICI in NPH, CD, and hypothyroidism. Moreover, the progression of HE from minimal (without a clear cognitive impairment) to an overt HE can be seen as an example of how differentiate the TMS correlates of a disease from a dementing process in the context of that disease: basically normal TMS profile in minimal HE (normal rMT, normal or slightly increased CMCT) and clearly altered in overt HE (increased rMT, prolonged CMCT). Therefore, these neurophysiological measures might add useful information even in secondary dementia, especially when combined with suggestive clinical features and other diagnostic exams.

Finally, the possibility to detect cortical dysfunction and maladaptive plasticity, to monitor the disease course and complications, to probe the response to treatment, including innovative neuromodulatory interventions, still represents a gap in the literature and a challenge for research. In this scenario, the identification of reliable TMS metrics in secondary dementia will provide not only biomarkers useful for the diagnosis and the prognosis, but also possible targets for non-invasive neuromodulation protocols. Indeed, the possibility to detect cortical dysfunction and maladaptive plasticity will greatly benefit from the recording of pattern consistently altered in specific conditions, that can be better evaluated in experimental models and then possibly applied also to probe the response to pharmacological or neuromodulatory treatments in longitudinal studies. As such, in addition to the already available studies, which have used only high-frequency (excitatory) rTMS in a few disorders (i.e., chronic ethanol abuse, MS, and SD-related cognitive impairment), other protocols using low-frequency (inhibitory) or high-frequency protocols customized on the TMS patterns observed should be prompted. For instance, low frequency rTMS might be applied to reduce hyperexcitability in some disorders (e.g., reduced rMT and SICI in NPH; reduced SICI and cSP and increased ICF in CD), whereas further high frequency protocols might be considered in others with increased rMT and prolonged CMCT (e.g., HE, OSAS, and SCA2).

The research agenda within these promising lines of investigation may include the following:

(i) defining reliable TMS parameters of cortical circuitry dysfunction; this will require to focus on single or pooled measures that show to be consistently altered in a given disease typically associated with cognitive impairment, in order to overcome the issue of undersized studies;
(ii) obtaining further neurophysiological insights on the pathophysiological changes typical of each disorder considered, that might be investigated in experimental models and tested as a target of new pharmacological treatments or neuromodulatory interventions;
(iii) expanding the exploration of functional response to therapies; this applies to all potentially treatable or modifiable conditions and will require longitudinal trials monitoring the course of cognitive impairment and TMS indexes of cortical plasticity or cortico-spinal conductivity.

## Author contributions

GL and VD: conceptualization. FF and MC: literature search. RD and FR: data analysis. GL and AC: writing – original draft preparation. MP and AG: writing – review and editing. RB and VD: supervision. All authors contributed to the article and approved the submitted version.
